# F26. THE NATURE OF CLINICAL HIGH-RISK SYMPTOMS: NEW INSIGHTS GAINED FROM AGE EFFECTS

**DOI:** 10.1093/schbul/sby017.557

**Published:** 2018-04-01

**Authors:** Frauke Schultze-Lutter, Chantal Michel, Stefanie Schmidt

**Affiliations:** 1 Heinrich-Heine University Düsseldorf; 2 University Hospital of Child and Adolescent Psychiatry, University of Bern

## Abstract

**Background:**

Early detection of psychosis is an important topic in psychiatry and is involving ever younger patient groups. Yet, developmental issues are still under researched. Thus, we examined risk symptoms and criteria in 8-40-year-olds from the general population.

**Methods:**

Clinical high-risk symptoms, i.e. attenuated and transient psycvhotic symptoms (APS, BIPS) as well ascognitive and perceptive basic symptoms (BS), were assessed by well-trained psychologists performed assessments of risk symptoms, using established interviews. Differentiating between perceptive and non-perceptive/cognitive phenomena, impact of age groups on risk symptoms and their clinical significance (current psychosocial functioning deficits or non-psychotic DSM-IV axis-I disorder) was assessed by logistic regression analyses.

**Results:**

Altogether, 9.9% of interviewees (N=689) reported APS, and 18.1% BS; 1.3% met APS, 3.3% COPER and 1.2% COGDIS criteria. For APS, an age effect was detected around age 16: compared to 16-40-year-olds, 8-15-year-olds reported more perceptive APS and lesser clinical significance of non-perceptive APS. Similar age effects of BS on prevalence and clinical significance that differed between perceptive and cognitive BS and followed brain maturation patterns were also detected: around age 18 for perceptive and in the early twenties for cognitive BS.

**Discussion:**

These findings strongly suggest differential developmental factors affecting prevalence and clinical significance of APS and BS: While neurocognitive maturation might influence the presence of APS, brain maturation seems to influence the presence of BS. These findings emphasize the need to address the differential effects of perceptive and non-perceptive risk phenomena, and their interaction with age, also in terms of conversion to psychosis, in future studies.

